# Exploratory Use of Proximal Cryoneurolysis and Distal Botulinum Toxin Type A for Upper-Limb Spasticity: A Case Report with Scoping Review

**DOI:** 10.3390/toxins18020066

**Published:** 2026-01-27

**Authors:** Luigi Di Lorenzo, Bruno De Meo, Alfonso Maria Forte, Francesco Forte, Vincenzo Palmieri, Nicola Pirraglia, Carmine D’Avanzo

**Affiliations:** 1 Department Vita, Salute e Professioni Sanitarie, Università Campus Link, 00165 Rome, Italy; 2 Rehabilitation and Pain Medicine I.N.M., Neuromed IRCCS, 86077 Pozzilli, Italy; 3 Biomedical Research Center, Gruppo Forte, 84124 Salerno, Italy

**Keywords:** cryoneurolysis, upper-limb spasticity, botulinum toxin type A, shoulder girdle, neurorehabilitation

## Abstract

Background: Upper-limb spasticity involving the shoulder girdle and elbow flexors often impairs functional hand use, and although Botulinum toxin type A (BoNT-A) is a first-line therapy, severe proximal synergies may persist while higher doses risk distal weakness. Methods: We report a case of a 47-year-old woman with neurodegenerative tetraparesis and marked shoulder and elbow flexor spasticity treated with bilateral percutaneous cryoneurolysis of the lateral pectoral, thoracodorsal, and musculocutaneous nerves, followed by distal BoNT-A injections, and we conducted a scoping review following Arksey and O’Malley, Levac, and PRISMA-ScR methods to contextualize the current evidence. Results: At one-month follow-up, the patient showed a reduction in MAS from 4 to 1–2, complete resolution of pain, improved passive shoulder abduction and elevation, preserved distal dexterity, and high satisfaction with no adverse events. The scoping review identified consistent MAS and range-of-motion improvements across multiple case reports and small series involving similar proximal nerve targets. Conclusions: The combined proximal cryoneurolysis–distal BoNT-A approach appears to be a feasible dual-modulation strategy for complex upper-limb spasticity when the preservation of hand function is essential, and the emerging literature supports its further investigation.

## 1. Introduction

Upper-limb spasticity is a frequent consequence of upper motor neuron lesions and may affect up to 40–50% of patients within the first year after stroke [1,2]. Shoulder girdle involvement is particularly disabling, as it contributes to pain, abnormal postures, and hygiene difficulties and limits the ability to position the hand in the functional workspace [1,3].

Botulinum toxin type A (BoNT-A) is widely accepted as a first-line pharmacological therapy for focal and multifocal spasticity and is strongly supported by randomized controlled trials and international consensus guidelines [1,2,4]. Recent expert statements emphasize a patient-centered, goal-oriented, multidisciplinary approach, with flexible dosing and repeated injection cycles tailored to individual patterns and priorities [3,4].

Despite this robust evidence base, the shoulder muscles are less frequently injected with BoNT-A than the elbow, wrist, and finger flexors, although trials and observational studies have demonstrated clinically relevant improvements in pain, range of motion, and function when the pectoralis major, teres major, subscapularis, latissimus dorsi, and related muscles are appropriately targeted [2,3]. The practical guide by Jacinto et al. has recently systematized shoulder patterns and recommended injection strategies according to two main patterns (A and B) of shoulder spasticity, reinforcing the importance of proximal pattern control to enable distal hand use [3].

However, even with optimized BoNT-A treatment, complex shoulder and elbow synergies may remain partially refractory, especially in patients with long-standing spasticity or fixed components, or those who cannot tolerate higher cumulative toxin doses. In this context, there is growing interest in integrating BoNT-A with non-pharmacological interventions (e.g., stretching, casting, electrical stimulation, orthotics, and other physical modalities), as advocated for by major guidelines and umbrella reviews [2,4].

Percutaneous cryoneurolysis has recently emerged as a minimally invasive neuromodulatory technique that applies extremely low temperatures to peripheral motor branches, producing a reversible Wallerian-like axonotmesis (temporary axonal disruption with preserved connective sheaths, followed by regeneration) with subsequent regeneration over weeks to months. A growing body of case reports, case series, and observational studies has described cryoneurolysis for upper- and lower-limb spasticity, including in the musculocutaneous, pectoral, thoracodorsal, and subscapular nerves, with consistent reductions in MAS scores, an increased range of motion, and high patient satisfaction, with a low incidence of serious adverse events [5,6,7,8,9,10,11,12,13,14,15,16,17].

The present article has two aims: (1) to describe a case in which bilateral proximal cryoneurolysis of shoulder girdle and elbow flexor nerves was strategically combined with distal BoNT-A injections in order to reduce pathological synergies while preserving essential hand function and (2) to provide a case-based scoping review of the literature on cryoneurolysis for upper-limb spasticity, with particular attention to the emerging “proximal cryo–distal BoNT-A” paradigm.

### Case Report

A 47-year-old woman with a progressive neurodegenerative disorder presented with tetraparesis and severe upper-limb spasticity. The patient presented with a long-standing, slowly progressive spastic tetraparesis, beginning in early adolescence. The initial symptoms emerged between the ages of 12 and 14, as progressive gait difficulty characterized by a sensation of lower-limb heaviness, followed over the years by loss of independent ambulation and the development of dysarthric speech. Since 1999, she has been treated with continuous intrathecal baclofen via implanted pump, currently delivering 400 μg/day, with partial control of generalized spasticity.

The clinical phenotype was characterized by predominant upper motor neuron involvement, including severe spasticity of all four limbs, progressive loss of ambulation, dysarthria, oculomotor abnormalities, and preserved cognitive function.

Extensive diagnostic investigations performed over several years at tertiary neurological centers, including neuroimaging, neurophysiological studies, cerebrospinal fluid analysis, metabolic screening, and broad genetic testing, did not identify a specific biochemical or genetic etiology. Neurophysiological findings consistently supported predominant upper motor neuron involvement with corticospinal tract dysfunction. Based on the progressive clinical course, exclusion of alternative causes, and multidisciplinary neurological evaluation, the condition was classified as a complex hereditary spastic paraplegia-like disorder. Over the years, the disease followed a slowly progressive course, despite continuous rehabilitation, intrathecal baclofen therapy, prior selective neurotomies of the lower limbs, and repeated cycles of botulinum toxin injections at approximately four-month intervals.

Over the past two decades, the patient has undergone continuous neuromotor rehabilitation, including regular physiotherapy and speech therapy, with the goal of preserving posture, joint mobility, and residual motor control. In addition to intrathecal baclofen therapy, she has received repeated cycles of botulinum toxin injections approximately every four months to manage focal spasticity, particularly in the lower limbs. Despite these long-term interventions, the progression of spasticity—especially at the shoulder girdle and elbow flexors—led to significant impairment in positioning, hygiene, and passive range of motion, motivating the consideration of an integrated focal interventional strategy.

Clinically, she exhibited marked spasticity of the shoulder girdle muscles and elbow/forearm flexors, with modified Ashworth scale (MAS) scores of 3–4, painful passive stretch, and substantially reduced passive and active range of motion. The pattern was dominated by shoulder adduction, internal rotation, and flexion, with fixed flexor posturing of the elbow and forearm.

A grayscale pencil-style illustration of the patient shows the typical upper-limb spasticity posture: shoulder adduction and internal rotation, moderate elbow flexion, wrist flexion, and synergistic finger flexor overactivity that is more pronounced on the left side. The drawing highlights preserved voluntary control of the first three digits of the right hand, allowing for computer use, despite proximal spasticity. Illustrative markers indicate the proximal targets treated with ultrasound-guided cryoneurolysis (lateral, pectoral, and thoracodorsal nerves at the shoulder girdle) and the distal muscles treated with botulinum toxin type A (brachioradialis, flexor carpi radialis, and finger flexors). The baseline assessment included quantitative goniometric evaluation of the shoulder’s range of motion and pain assessment using a visual analogue scale (VAS), distinguishing pain at rest and during movement. Standardized functional outcome measures (e.g., DASH, ARAT) were not formally collected at the baseline, representing a limitation of this case report. Distal voluntary motor control of the fingers was preserved, based on qualitative clinical examination (Table 1). At the baseline, distal motor function was severely impaired. The patient was able to perform limited voluntary forearm pronation–supination (approximately 10–20°), slow wrist flexion–extension (10–20°), and minimal digit movement involving the first and second fingers in partial flexion. No functional grasp, grip, or pinch was present. Alternative approaches such as goal attainment scaling or caregiver-rated measures may represent feasible options in future similar cases.

Despite the severity of her proximal spasticity, the patient retained critical voluntary control of the first two digits of her dominant hand, which she used to type on a computer keyboard and manage environmental controls. Preservation of this distal functional capacity was identified as a priority goal by the patient, caregivers, and rehabilitation team.

**Figure 1:**
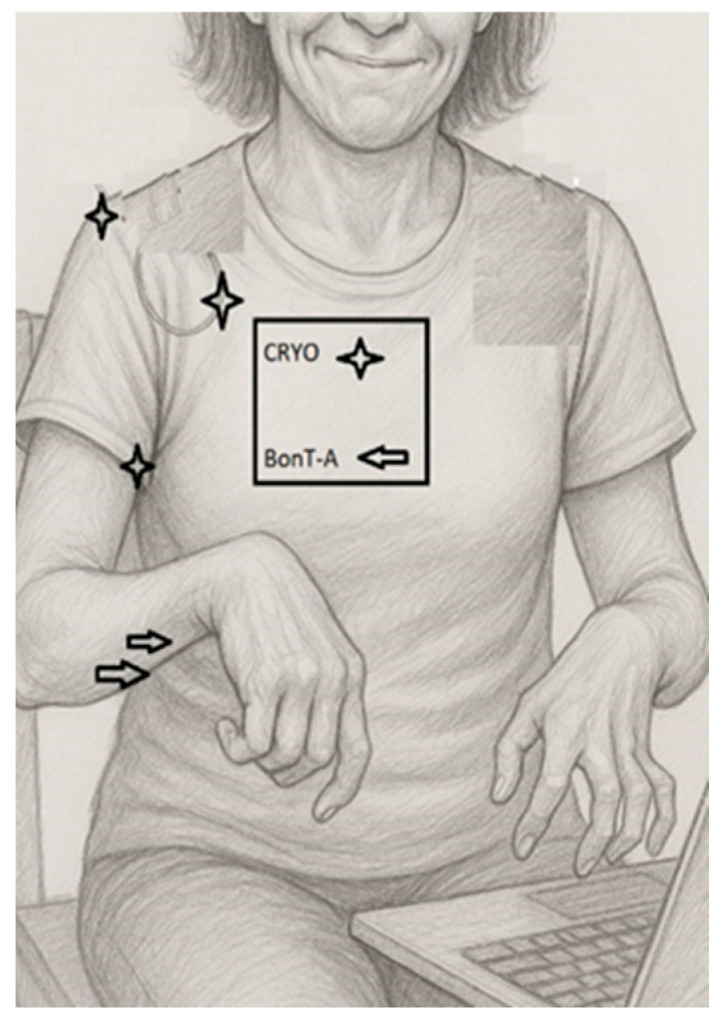
Clinical–functional illustration of upper-limb spasticity pattern and treatment targets.

Given the limited benefit of previous pharmacological treatments and the desire to avoid diffuse distal weakness, a combined interventional strategy was planned. Ultrasound-guided bilateral cryoneurolysis was performed, targeting: (1) the lateral pectoral nerve supplying the pectoralis major, (2) the thoracodorsal nerve innervating the latissimus dorsi, and (3) the musculocutaneous nerve supplying the biceps brachii and brachialis (Figure 1). The goal was to modulate the pathological shoulder adduction–internal rotation and elbow flexor synergies at their proximal “drivers” while sparing more distal motor units.

## 2. Procedural Technique

The musculocutaneous nerve was identified under ultrasound guidance, using a high-frequency linear probe (GE Healthcare ultrasound system). Initial localization was performed at the axillary level, using the axillary artery as an anatomical landmark, as is commonly adopted for brachial plexus blocks. The nerve was then traced distally for several centimeters until it was visualized as coursing between the biceps brachii and the brachialis muscles, which represented the target site for cryoneurolysis.

Nerve identification was further confirmed using the integrated nerve stimulation module of the cryotherapy system, eliciting a consistent motor response that was compatible with musculocutaneous nerve activation.

**Figure 2:**
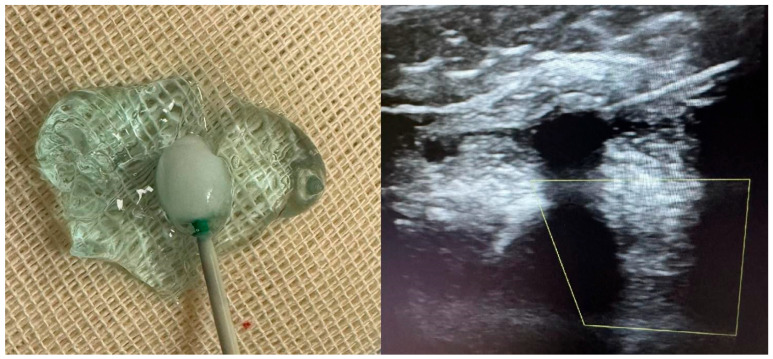
Ultrasound-guided cryoneurolysis: ice-ball formation and probe visualization. (**left**) The cryoprobe tip producing a stable ice-ball in conductive gel immediately after activation, illustrating the spherical zone of the endothermic tissue interaction that corresponds to the expected lesion geometry. (**right**) Real-time ultrasound image during cryoneurolysis of a proximal upper-limb motor nerve. The hypoechoic circular region surrounding the probe represents the developing ice-ball, which encases the target nerve while sparing adjacent structures. This corresponds to the reversible axonotmesis mechanism underlying cryoneurolysis.

Cryoneurolysis was performed, using a dedicated cryotherapy system (Cryo-S Painless, Metrum Cryoflex, Łomianki, Poland) connected to sterile, single-use cryoprobes supplied within the “Kit Lesione Crio” (CDF Medical Solutions S.r.l., Camaiore, Italy). An 18-gauge cryoprobe was employed. The system operates with medical-grade CO_2_ gas under pressure (600–800 psi), generating a closed-circuit Joule–Thomson effect at the probe tip and producing an ice ball (Figure 2) with a target temperature of approximately −70 °C. The target temperature (≈−70 °C) refers to device specifications; temperature was not directly measured in tissue. Ice-ball formation was visualized under ultrasound; its diameter was not formally measured.

Cryoapplication was delivered according to the manufacturer’s specifications, with ultrasound visualization of ice-ball formation at the probe tip. The closed-circuit system ensured that the gas did not come into direct contact with patient tissues. Cryoneurolysis induces a temporary inhibition of nerve conduction while preserving neural and surrounding connective structures, with expected spontaneous functional recovery over several months.

Local anesthesia was administered at the skin entry site. The patient neither required nor requested procedural sedation, and the procedure was completed under local anesthesia alone without complications

## 3. Botulinum Toxin Injection Protocol

Following cryoneurolysis, botulinum toxin type A was administered distally to target residual spasticity while preserving distal voluntary motor control. OnabotulinumtoxinA (Botox^®^, AbbVie, Chicago, IL, USA) was used, with a total dose of 200 IU diluted in 1 mL of saline solution (Table 2). Injections were performed approximately 30 min after completion of the cryoneurolysis procedure. BoNT-A injections were performed ~30 min after cryoneurolysis for procedural logistics and short post-procedural observation; no specific physiological rationale was assumed.

The following muscles were treated: flexor digitorum profundus (75 IU in two ultrasound-guided injection points), flexor digitorum superficialis (50 IU in two ultrasound-guided injection points), pronator teres (50 IU in one ultrasound-guided injection point), and flexor carpi radialis (25 IU in one anatomically guided injection point). Ultrasound guidance was employed to optimize accuracy and minimize diffusion, except for the flexor carpi radialis, which was injected using anatomical landmarks [16].

## 4. Outcome Assessment and Follow-Up

Clinical outcomes were assessed at one and two months after the procedure. At the two-month follow-up, the patient’s clinical status was substantially unchanged compared with the one-month evaluation, with maintenance of the observed improvements in proximal range of motion, pain during movement, and muscle tone.

The expected duration of the cryoneurolysis effect, based on the mechanism of action and the available literature, ranges from approximately 8 months to 1 year, corresponding to the time required for axonal regeneration and recovery of nerve conduction.

A structured follow-up plan was established. A further clinical evaluation was scheduled at three months, at which time additional cryoneurolysis treatment was planned for other spastic muscle groups, specifically the hip adductors and the sural region, according to the patient’s overall spasticity distribution.

Distal motor function was described through quantitative range-of-motion measurements and qualitative clinical examination (Table 1). Although distal dexterity was preserved and subjectively improved, standardized functional outcome measures could not be administered. This limitation was primarily related to the patient’s inability to communicate verbally; the patient relies on a computer-based communication device, which limited the feasibility of validated self-reported and performance-based functional assessments.

One month after the combined procedure, the patient provided written feedback. The patient wrote: “more loose and relaxed, free from pain… my hands feel softer and easier to treat”. She denied any adverse effects; expressed a desire to receive the same treatment for her lower limbs; and asked for the name of the scientific journal in which the results would be published. Specifically, the patient explicitly requested cryoneurolysis for the hip adductors, the posterior tibial compartment and the triceps surae, stating that the improvement achieved in the upper limb convinced her of the potential usefulness of extending the treatment to the lower limbs as well. Clinically, the MAS scores decreased from 4 to 1–2 at the shoulder and elbow. Pain upon passive stretching completely resolved, abduction and elevation of the shoulder noticeably improved, and the arm became easier to position for hygiene, dressing, and transfers. Distal dexterity, grasp–release capability, and the ability to type on a keyboard were preserved and subjectively improved, due to the reduction in proximal co-contraction. No complications or sensory adverse events were reported during follow-up.

## 5. Results

The search identified a focused but growing body of literature on percutaneous cryoneurolysis for limb spasticity, including several reports specifically addressing the upper limb and shoulder girdle. The most substantial evidence comes from a 2019 case series by Winston and colleagues, describing cryoneurotomy as a percutaneous, minimally invasive therapy for spastic limbs, involving both upper and lower extremities and including musculocutaneous and other motor nerves; significant improvements in MAS scores and range of motion were reported, with acceptable safety [5]. Rubenstein and Shah illustrated the use of cryoneurotomy of the musculocutaneous nerve to reduce flexed-elbow spasticity, demonstrating functional gains in a visual vignette [6]. A case study presented by Jorgensen and Vincent at the Toxins Society meeting reported cryoneurotomy of a branch of the musculocutaneous nerve as a novel adjunct to BoNT-A in a patient with post-stroke flexed-elbow spasticity after multiple prior toxin cycles, enabling the redistribution of BoNT-A to distal finger flexors while improving the elbow’s range of motion [7]. David et al. described multisite percutaneous cryoneurolysis in adults with long-standing post-stroke spasticity, treating both upper- and lower-limb regions, including shoulder adductors, and documenting reductions in the MAS and an improved range of motion [8]. Hashemi Taheri et al. conducted a prospective observational study of 59 adults with plateaued or refractory shoulder spasticity after prior BoNT-A, showing that cryoneurolysis of upper-limb motor nerves (including those supplying the shoulder) produced sustained improvements in spasticity, function, and patient satisfaction for up to 12 months [9]. Winston et al. evaluated percutaneous cryoneurolysis for pain in patients with upper-extremity spasticity, reporting reductions in pain intensity and enhanced passive and active range of motion, particularly involving the shoulder region [10]. Guynn and Winston presented a case series of ten patients with upper- and lower-limb spasticity treated with percutaneous cryoneurolysis of multiple nerves, including pectoral, thoracodorsal, and subscapular branches, confirming that the technique is a dynamic and adaptable treatment associated with meaningful reductions in spasticity and improvements in range of motion, with no serious complications [11]. Hughes et al. reported a case of multiple sclerosis-related spasticity treated with cryoneurolysis across multiple segments including the shoulder, observing improvements in tone and multi-district mobility [12]. Mumby and co-authors described cryoneurolysis combined with factor VIII administration in a young patient with cerebral palsy and hemophilia A who had elbow and wrist flexor spasticity refractory to BoNT-A; cryoneurolysis improved tone and range of motion without bleeding complications [13]. Herzog et al. presented a case of severe post-arteriovenous malformation shoulder spasticity and long-standing contracture, treated with combined cryoneurolysis of proximal shoulder nerves and percutaneous needle tenotomy, leading to major gains in range of motion and ease of care [14].

At the level of evidence synthesis, Winston published a scoping review summarizing cryoneurolysis as a novel treatment for spasticity, highlighting consistent efficacy signals across small heterogeneous studies and mentioning historical data on obturator nerve cryoablation [15]. Additional pilot data from Pacira BioSciences report preliminary efficacy of percutaneous cryoneurolysis for wrist, hand, and finger spasticity, as well as for spasticity-related pain in upper-limb muscles, reinforcing the concept of using cryoneurolysis along the proximal–distal continuum of the upper limb [16]. ClinicalTrials.gov lists ongoing trials (NCT04670783, NCT06782464) investigating cryoneurolysis of upper-limb nerves for spasticity, including comparisons with usual care, although the results are not yet available [17].

When the literature is examined through the lens of shoulder girdle involvement, several reports converge on the same anatomical targets as those recommended for BoNT-A injection in expert guidelines: notably, the pectoralis major, teres major, latissimus dorsi and subscapularis muscles, and their innervating nerves [1,2,3]. The series by Guynn, David, and Hashemi Taheri collectively demonstrate that cryoneurolysis of the lateral pectoral, thoracodorsal, and related branches can safely reduce shoulder adduction–internal rotation patterns, improve hygiene, dressing, and transfers, and relieve pain in patients with long-standing post-stroke or other acquired spasticity [8,9,11].

Across studies, BoNT-A is often present in the background treatment history: many patients had received multiple prior toxin cycles, with cryoneurolysis considered when responses plateaued or distal weakening became a concern [5,8,9]. This parallels the broader spasticity literature, where umbrella reviews and consensus guidelines recommend combining pharmacological and non-pharmacological strategies within an individualized rehabilitation program, rather than relying on a single modality [2,4]. However, with the exception of the Jorgensen and Vincent case study [3], no report has yet formalized a protocol in which proximal cryoneurolysis is purposefully paired with planned distal BoNT-A injections to preserve specific voluntary functions, such as hand use for typing or grasp–release tasks. Our case directly addresses this gap (Table 3).

## 6. Discussion

This case illustrates how proximal cryoneurolysis and distal BoNT-A can be combined to achieve dual modulation of complex upper-limb spasticity in a patient who still relies on residual hand function for meaningful daily activities. By selectively targeting the lateral pectoral, thoracodorsal, and musculocutaneous nerves, cryoneurolysis effectively reduced the pathological shoulder adduction–internal rotation and elbow flexion synergies that interfered with posture, comfort, and care, while sparing distal motor units that were essential for fine hand control. Distal BoNT-A injections were then used as a “fine-tuning” tool to modulate residual focal over-activity in the forearm and finger flexors without compromising the preserved digits.

This approach is coherent with current expert recommendations for the management of shoulder spasticity, which emphasize patient-centered goal setting, careful analysis of movement synergies and prioritization of muscles that hinder functionally relevant activities [1,3,4]. In Jacinto et al.’s guide, the primary role of the shoulder is to position the hand within the functional workspace; therefore, treating shoulder patterns that restrict reaching and hygiene is a prerequisite for effective distal rehabilitation [3]. Our case operationalizes this concept by using cryoneurolysis as a proximal “reset” of pathological synergies, followed by a BoNT-A-based refinement of distal tone.

Consensus statements from Francisco and colleagues further support a flexible, individualized BoNT-A strategy, including dose adjustments, repeated cycles, and the integration of adjunctive therapies such as stretching, casting, orthoses, and advanced neuromodulation techniques [4]. Within this framework, cryoneurolysis can be viewed as an adjunctive focal intervention aimed at changing the biomechanical context in which BoNT-A operates: by reducing the “driver” activity of proximal muscles, it may allow for lower doses of toxin to be used distally, potentially minimizing unwanted weakness and extending the functional benefits of each treatment cycle. Given the longer expected duration of cryoneurolysis effects (approximately 8–12 months) compared with typical BoNT-A reinjection intervals (around 3–4 months), this combined approach may allow for staged or complementary treatment cycles. In this context, cryoneurolysis may act as a proximal reset of pathological synergies, while BoNT-A can be used for interim distal fine-tuning according to functional goals and clinical reassessment.

From a mechanistic standpoint, cryoneurolysis produces a temporary interruption of efferent motor signals and afferent feedback along selected nerves, followed by gradual reinnervation. This may provide a “therapeutic window,” during which rehabilitation, stretching, and task-oriented training can be intensified, taking advantage of reduced resistance and pain. Such a window is analogous to that described for BoNT-A in guidelines that stress the importance of pairing injections with structured rehabilitation and goal-oriented programs to maximize gains in activity and participation [1,4].

The literature summarized in our scoping review shows a consistent profile: across different nerve targets and patient populations, percutaneous cryoneurolysis is associated with meaningful improvements in tone, range of motion, and pain, with very few serious complications [5,6,7,8,9,10,11,12,13,14,15,16,17]. This aligns with a broader umbrella of review evidence, indicating that non-pharmacological interventions—including physical modalities, neuromodulation, and focal procedures—can complement pharmacological management to provide multi-dimensional benefits in spasticity [2]. Nevertheless, the existing studies are small, often uncontrolled, and heterogeneous in terms of techniques, outcome measures, and follow-up duration.

Our case adds several novel elements: (1) it focuses specifically on shoulder girdle and elbow flexor spasticity in a patient with a neurodegenerative, rather than purely post-stroke, condition; (2) it documents patient-reported outcomes highlighting pain relief, perceived relaxation, and high satisfaction, alongside objective MAS and range-of-motion improvements; and (3) it explicitly describes a treatment paradigm in which proximal cryoneurolysis is intentionally combined with distal BoNT-A to preserve an identified functional task (computer typing). To our knowledge, no previous series has systematically described this “proximal cryo–distal BoNT-A” strategy, making this case particularly relevant for clinicians seeking to reconcile tone reduction with functional preservation.

Several limitations must be acknowledged. As a single case, our observations cannot establish causality or generalizability. The neurodegenerative etiology and the specific pattern of residual function may not mirror typical post-stroke presentations. Post-stroke spasticity represents the most common clinical context in which shoulder and elbow flexor spasticity is encountered, and the treatment responses, recovery trajectories, and functional priorities may differ from those observed in slowly progressive neurodegenerative conditions. Therefore, an extrapolation of these findings to post-stroke populations should be made with caution, despite the presence of shared biomechanical and synergy-related features. Quantitative measures of dexterity and upper-limb function were not systematically collected. In patients with severe communication impairments, alternative validated approaches such as goal attainment scaling or caregiver-rated outcome measures may provide more feasible assessments and should be considered in future studies. Finally, our scoping review, although comprehensive, relied on the published English-language literature, and may have missed unpublished or non-English data.

Despite these limitations, the convergence between our clinical experience and the emerging literature suggests that proximal cryoneurolysis deserves consideration as part of the interventional armamentarium for complex shoulder and elbow spasticity, particularly in patients in whom distal function is still meaningful and must be protected.

## 7. Conclusions

Combined proximal cryoneurolysis and distal BoNT-A represents a biologically plausible and clinically attractive dual-modulation strategy for complex focal spasticity of the upper limb when preservation of distal movement is essential. In the presented case, bilateral cryoneurolysis of lateral pectoral, thoracodorsal, and musculocutaneous nerves, followed by targeted BoNT-A injections into distal flexor muscles, yielded marked reductions in MAS, elimination of pain, improved shoulder range of motion, and maintenance of critical hand function, with excellent patient-reported satisfaction and no adverse events.

The accompanying case-based scoping review shows that percutaneous cryoneurolysis of proximal upper-limb nerves is consistently associated with reductions in muscle tone, improvements in range of motion, and good tolerability across heterogeneous clinical contexts [17]. While this combined approach may represent a promising avenue for managing refractory shoulder spasticity, it must be interpreted as hypothesis-generating only. Given the single-case design and short-term follow-up, no conclusions can be drawn regarding efficacy or generalizability. Prospective studies with standardized functional outcomes and longer observation are warranted [14,15,16,17]. This case report, while clinically informative, is limited by its anecdotal nature, lack of standardized functional assessments, and short-term follow-up. Findings should be interpreted cautiously and cannot support definitive conclusions regarding efficacy or generalizability. Prospective controlled studies are warranted to define optimal patient selection, nerve targets, timing relative to BoNT-A, and the long-term functional outcomes of this combined approach.

## 8. Methods

### Literature Analysis

Before the case management, we conducted a scoping review of the available literature, following the methodological principles outlined by Arksey and O’Malley and refined by Levac and collaborators, in accordance with the PRISMA-ScR reporting standards [18,19,20]. The objective was to map all existing evidence concerning percutaneous cryoneurolysis, alone or combined with BoNT-A, for upper-limb spasticity of the shoulder girdle and forearm.

A structured literature search was performed in PubMed/MEDLINE, Scopus, and Web of Science, covering studies published from January 2020 to January 2025. The search strategy combined free-text terms related to cryoneurolysis and spasticity, including the following: cryoneurolysis, cryoneurotomy, cryotherapy, spasticity, upper limb, shoulder, elbow, forearm, hand, and botulinum toxin.

Titles and abstracts were independently screened by two reviewers, followed by full-text assessment of potentially eligible articles. Disagreements were resolved through discussion and consensus with the senior author.

Data extraction included the following: study design, year of publication, sample size, neurological condition, target nerve(s), anatomical region treated, use of concomitant therapies (including BoNT-A), outcome measures (e.g., MAS, range of motion, pain, functional outcomes), follow-up duration, and reported adverse events.

In line with PRISMA-ScR recommendations, and given the exploratory nature of the available literature, no formal quality or risk-of-bias assessment was performed. The review was not registered (Figure S1, Flow Diagram).

Reference lists of all eligible articles were manually screened to identify additional sources.

Eligibility criteria were deliberately broad. We included human studies of any design (case reports, case series, observational cohorts, pilot trials) in which percutaneous cryoneurolysis was employed to treat upper-limb spasticity and in which at least one proximal neural target that was relevant to the shoulder girdle or elbow flexors was addressed (e.g., lateral/medial pectoral nerves, thoracodorsal nerve, subscapular branches, musculocutaneous nerve). Studies integrating cryoneurolysis with BoNT-A were eligible when they reported at least one clinical outcome, such as MAS changes, range of motion, pain scores, functional measures, or adverse events. Only English-language articles were included for feasibility reasons, which may have limited the completeness of the evidence base. We excluded animal studies, open surgical cryoablations, reports focused purely on neuropathic pain or neuroma without spasticity, and interventions based exclusively on pharmacological therapy.

Two reviewers independently screened titles and abstracts and then assessed the full texts of potentially eligible studies. Discrepancies were resolved through discussion with the senior author. Data extraction captured patient characteristics, anatomical targets, technical aspects of cryoneurolysis, concomitant treatments (including BoNT-A), outcome measures, follow-up duration, and safety. Because the review synthesized previously published data, no ethical approval or additional patient consent was required.

Given the early developmental stage of cryoneurolysis for spasticity and the predominance of non-randomized evidence, a scoping review design was chosen to provide a comprehensive mapping of the current knowledge, identify recurring neuroanatomical targets and therapeutic patterns, and highlight knowledge gaps to inform future controlled trials.

This scoping review was conducted following the methodology of Arksey and O’Malley and Levac et al. and is reported in accordance with the PRISMA-ScR (Preferred Reporting Items for Systematic Reviews and Meta-Analyses extension for Scoping Reviews) guidelines.

## Figures and Tables

**Table 1:** Baseline and follow-up clinical assessment.

Domain	Measure	Baseline (Pre-Cryo)	Post-Procedure	1-Month Follow-Up	2-Month Follow-Up
Shoulder ROM	Flexion (°)	20°	50°	50°	50°
Shoulder ROM	Internal–External rotation (°)	20°	40–50°	40–50°	40–50°
End-feel	Passive resistance at end-range	Markedly increased elastic resistance	Improved	Maintained improvement	Maintained improvement
Shoulder girdle elasticity	Passive and active elasticity	Severely reduced	Improved	Maintained	Maintained
Pain (VAS)	At rest	0–2	0–2	0–2	0–2
Pain (VAS)	During movement	7–8	3–4	3–4	3–4
Distal motor control	Forearm pronation–supination	10–20° (slow, voluntary)	Preserved	Preserved	Preserved
Distal motor control	Wrist flexion–extension	10–20° (slow)	Preserved	Preserved	Preserved
Distal motor control	Fingers	Minimal voluntary movement of first and second digits in partial flexion; no functional grasp, grip, or pinch	Preserved	Preserved	Preserved
Functional outcome	DASH/ARAT	Not formally assessed	—	—	—

Baseline (Pre-Cryo) refers to assessments performed before the cryoneurolysis procedure. Post-Procedure indicates evaluations conducted immediately after the intervention. Follow-up assessments at 1 and 2 months were performed to monitor the short- and mid-term clinical effects and durability of treatment outcomes. End-feel and shoulder girdle elasticity represent qualitative clinical assessments based on passive examination.

**Table 2:** Botulinum toxin type A injection protocol.

Muscle	Abbreviation	Dose (IU)	Injection Points	Guidance Technique
Flexor digitorum profundus	FDP	75 IU	2 points	Ultrasound-guided
Flexor digitorum superficialis	FDS	50 IU	2 points	Ultrasound-guided
Pronator teres	PT	50 IU	1 point	Ultrasound-guided
Flexor carpi radialis	FCR	25 IU	1 point	Anatomical (non–ultrasound-guided)
Total dose		200 IU		

**Table 3:** Summary of studies included in the scoping review on percutaneous cryoneurolysis for upper-limb spasticity.

Author, Year	Study Design	Sample Size	Population/Etiology	Target Nerves/Regions	Main Outcomes Reported	Follow-Up	Reported Adverse Events
[5].	Case series	Not specified	Upper and lower limb spasticity (mixed etiologies)	Musculocutaneous and other motor nerves	↓ MAS, ↑ ROM	Not specified	No serious ones reported
[6].	Case report (visual vignette)	1	Spastic flexed elbow	Musculocutaneous nerve	↓ elbow spasticity, ↑ ROM	Not specified	Not reported
[7].	Case report (conference poster)	1	Post-stroke elbow flexor spasticity	Musculocutaneous nerve	↓ spasticity, redistribution of BoNT-A to distal muscles	Not specified	Not reported
[8].	Case report	1	Long-standing post-stroke spasticity	Multiple upper- and lower-limb motor nerves, including shoulder	↓ MAS, ↑ ROM	Not specified	Not reported
[9].	Prospective observational study	59	Plateaued or refractory shoulder spasticity (post-stroke)	Upper-limb motor nerves supplying shoulder	↓ spasticity, ↑ function, ↑ patient satisfaction	Up to 12 months	No serious adverse events reported
[15].	Observational study	Not specified	Upper-extremity spasticity with pain	Upper-limb motor nerves (shoulder region included)	↓ pain, ↑ passive and active ROM	Not specified	Not reported
[11].	Case series	10	Upper- and lower-limb spasticity	Pectoral, thoracodorsal, subscapular, and other motor branches	↓ spasticity, ↑ ROM	Not specified	No serious ones reported
[12].	Case report	1	Multiple sclerosis-related spasticity	Multiple upper-limb regions, including shoulder	↓ tone, ↑ mobility	Not specified	Not reported
[13].	Case report	1	Cerebral palsy with hemophilia A	Elbow and wrist flexor nerves	↓ spasticity, ↑ ROM	Not specified	No bleeding or procedure-related complications
[14].	Case report	1	Severe post-AVM shoulder spasticity with contracture	Proximal shoulder nerves + needle tenotomy	↑ ROM, ↑ ease of care	Not specified	Not reported

↓ = reduction; ↑ = increase; MAS = Modified Ashworth Scale; ROM = Range of Motion.

## Data Availability

The original contributions presented in this study are included in the article/Supplementary Material. Further inquiries can be directed to the corresponding author.

## Supplementary Materials

The following supporting information can be downloaded at: https://www.mdpi.com/article/10.3390/toxins18020066/s1, Figure S1: Flow Diagram.
