# Supplementary figures and images for: Exploratory Use of Proximal Cryoneurolysis and Distal Botulinum Toxin Type A for Upper-Limb Spasticity: A Case Report with Scoping Review
Source: Toxins (Basel). 2026 Jan 27;18(2):66. doi: 10.3390/toxins18020066 (PMC12944957; doi:10.3390/toxins18020066)

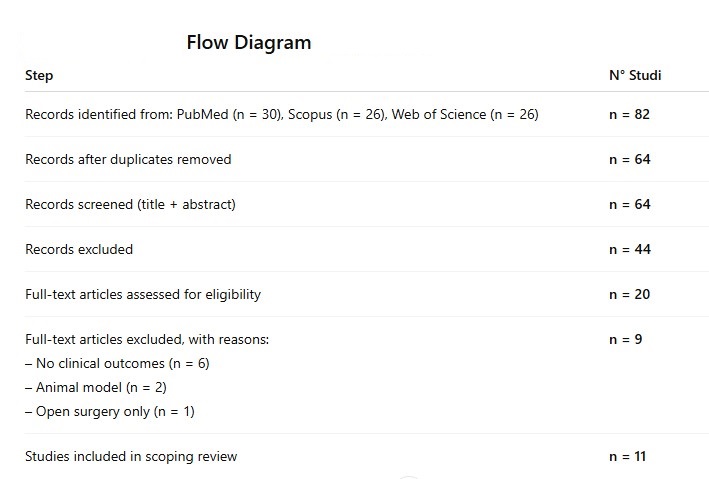

Supplement: Supplementary file 1 [file toxins-18-00066-s001.zip › toxins-4031410-supplementary.jpg]
